# Gaze cluster analysis reveals heterogeneity in attention allocation and predicts learning outcomes

**DOI:** 10.1038/s41598-025-06654-x

**Published:** 2025-06-25

**Authors:** Nathalie John, Sebastian P. Korinth, Mareike Kunter, Franziska Baier-Mosch

**Affiliations:** 1 Department of Psychology, Goethe University Frankfurt, Frankfurt am Main, Germany; 2 IDeA, Center for Individual Development and Adaptive Education of Children at Risk, Frankfurt am Main, Germany; 3 DIPF | Leibniz Institute for Research and Information in Education, Frankfurt am Main, Germany

**Keywords:** Eye tracking, Attention, Cluster detection, Learning, Instructional video, ISC, Psychology, Attention

## Abstract

Instructional videos need to maintain learners’ attention to foster learning, therefore, a fine-grained measurement of attention is required. Existing gaze measures like inter-subject correlation (ISC) assume a singular focal point deemed meaningful for indicating attention. We argue that multiple meaningful foci can exist and propose an automatically generated gaze measure labeled gaze cluster membership (GCM). By applying the density-based clustering in spatial databases (DBSCAN) algorithm to gaze position data from over 100 participants, we categorize viewers as attentive when they are part of a cluster and as inattentive when they are not. Using two videos, we demonstrate that our settings of DBSCAN generate meaningful clusters. We show that low ISC values (neuronal and eye tracking data) during multiple meaningful foci do not necessarily indicate a lack of attention. Additionally, GCM predicts participants’ self-reported mental effort and their tested knowledge. Our innovative approach is of high value for assessing learner attention and designing instructional videos.

## Introduction

Imagine you are watching a video, fully engaged in its content. You then discuss the video with a friend who has also watched it, and they mention that they missed important details and felt confused. This raises the question: Did your friend pay attention to the same elements of the video as you did? Did they pay attention at all?

Instructional videos have become an integral part of countless learning settings (e.g., massive open online courses (MOOCs))^1–3^. A development also boosted by the COVID-19 pandemic, for a large number of learners, online videos provide access to knowledge in a time- and location-independent manner and are often the primary means of knowledge transfer in post-secondary education^1,4^. Attention plays a crucial role in the learning process, serving as a key factor in determining which information is encoded into memory^5^. It directs our focus toward specific details, facilitating deeper processing and integration into our mental frameworks. Conversely, our internal models and prior knowledge also shape our attention, guiding what we notice and prioritize^5–7^. An obvious implication for the instructional design of such video is therefore the promotion of prolonged attention, which in turn should mitigate the high dropout rates typically seen in MOOCs^3^. In order to improve the quality and efficiency of their videos, instructors would benefit from information about which video sections are perceived as challenging, or when mind-wandering occurs among learners. Fine-grained measures of learners’ attention could provide this valuable information.

Existing approaches for measuring learners’ attention while watching videos can be categorized into retrospective and real-time measures^8^. Retrospective measures (e.g., self-reports) may be biased by memory effects and miss temporal variations^9^. Real-time measures like log data such as click stream interactions (e.g., pauses and replays)^10^ provide a better temporal resolution but are restricted to detecting behavioral manifestations of attention instead of measuring the cognitive state itself. In contrast, neurophysiological indicators of attention, such as neuronal oscillations in the alpha-band range obtained through electroencephalography (EEG) recordings, can be considered direct measures that provide the highest temporal resolution^11^. However, EEG methodology continues to pose challenges for implementation, particularly due to issues such as motion artifacts and the complexities involved in interpretation, and has so far mostly been studied in laboratory settings^12^.

A more accessible approach for measuring attentional states with high temporal resolution is eye tracking; that is, the capture of gaze position and movement, pupil size, and blink rates, which are typically achieved by means of videography^13–15^. On the one hand, eye tracking measures are driven by bottom-up visual dynamics (e.g., changes in scene lighting), which results in similar scan paths, even when videos are presented in scrambled order^16,17^. On the other, top-down processes like attention—also referred to as attentional state^18^ or attentional engagement^19^—affect eye movements, blink rate, and pupil size^18,19^.

Here, we will use the term attention, defined as the ability to direct and maintain focus on a specific object or area while selectively processing relevant information and filtering out irrelevant or distracting stimuli^20^. Following the eye-mind hypothesis—which postulates a robust correlation between eye movements and cognitive processes—visual attention can be interpreted as learners’ cognitive attention, because an individual’s visual focus reflects their mental activities with only minimal latency^21^. Nevertheless, the link between eye movements and attention allocation is certainly not perfect, as periods of mind-wandering for instance will disrupt the eye-mind connection^22^.

Evaluating and interpreting eye tracking data is particularly complex for dynamic stimuli such as videos, and various measures have been used in educational research. Changes in pupil size over time allow conclusions about attentional states^23^. Namely, stronger spontaneous fluctuation in pupil size can be observed when attention is decoupled from a task^24^. A difficulty in the interpretation of changes in pupil size is, however, separating cognitive top-down effects from those occurring due to variations in luminance of the presented stimuli or surrounding light conditions. Experimental tasks that require attention often reveal that blinking tends to be inhibited during these tasks, with instances of blinking being more likely to occur just before or after the task^25,26^.

Gaze position measures can also provide valuable insights into learners’ attention and are frequently assessed in relation to Areas of Interest (AOI), which are pre-defined (e.g., by the researcher) as the most relevant sections of a scene^13,14^. A disadvantage of AOI measures is, however, that their interpretability might be ambiguous. For instance, longer dwell times (i.e., the sum of all fixation durations in an AOI) may indicate more focused attention on the task^27^, but may also represent mind-wandering, that is, attention on other thoughts unrelated to the video^28^. In addition, the pre-defined nature of AOIs may introduce a bias towards video content that the researchers consider relevant and, most importantly, does not allow generalization to new video content.

In contrast, synchrony measures do not require any pre-defined assumptions and have been proposed as indicators of attention^18,29,30^. The core idea is that physiological signals become more similar among individuals when they attend the same stimuli. Similarity can be quantified through inter-subject correlation (ISC), which is based on correlated component analysis and attempts to detect time points in which data sets (e.g., gaze positions, EEG, variations in pupil size) show maximal correlation across subjects^18,31,32^. ISC was found to be higher when participants were attentively viewing a video compared to when they were distracted by a secondary task, such as counting backwards^18,29^. Furthermore, some researchers have found that these measures can be predictive for individual test scores on knowledge assessments^18,33^. In contrast, another study reported opposing results, revealing that the group with higher test scores exhibited lower inter-subject correlation^29^. Alternative methodologies to ISC for the measurement of gaze synchrony, such as the Kullback–Leibler Divergence of gaze density maps and Multi Match algorithm scan path comparisons, have been proposed but have not been found to be predictive for knowledge acquisition^30^.

The core premise of all gaze synchrony measures is the assumption of a single meaningful region that must be fixated to indicate attention. It is, however, plausible that various regions within a given scene convey meaningful information (e.g., conversation between several speakers^34^); fixations in each region may indicate attentive watching equally well. Established synchrony measures like ISC would falsely detect inattentiveness based on greater dissimilarity of gaze position data.

### The current study

To address this problem, we propose a new measure labeled gaze cluster membership (GCM). GCM is based on an unsupervised machine learning approach (i.e., DBSCAN^35,36^) that groups gaze position data into clusters at a single video frame level. Gaze positions of an individual belonging to one of the potentially several clusters were classified as attentive viewing. By contrast, inattentive viewing was detected when the gaze position of an individual could not be assigned to a cluster. The advantages of DBSCAN are the combination of automatic determination of the number of clusters and the flexible shape of the clusters^35,36^.

The current study seeks to validate and demonstrate the usefulness of GCM as an indicator of attention during video viewing, using two distinct videos: a snippet from the entertainment movie “Bang! You’re Dead” directed by Alfred Hitchcock (1961) and a self-produced instructional video called “Programming in Minecraft”. First, we want to demonstrate that the automatic cluster detection using DBSCAN reliably detects gaze clusters that provide relevant information for learning or for following the story in both videos. Second, to illustrate the potential problem single-focus measurements (e.g., ISC) encounter in multi-foci scenes, we contrast the ISC of the gaze data (ISC_Gaze_) and GCM with the cluster count for the video clip from “Bang! You’re Dead”. As the issue might not be limited to ISC_Gaze_ alone, given that various datasets (including gaze positions and EEG) exhibit correlations across subjects^31^, we also examine the ISC of the EEG data (ISC_EE__G_, ISC_Poulsen et al. 2017_). Third, to provide evidence that GCM can be interpreted as an indicator of attention, GCM should be associated with the related construct mental effort (i.e., how hard a person tries to actively process presented information^37^) in the instructional video. Fourth, to demonstrate the usefulness of GCM, we investigated whether it could predict posttest knowledge (controlling for prior knowledge) in the instructional video, as attention plays a crucial role in the learning process and the primary goal of instructional videos is to enhance learning^5–7,18,30^. Taking advantage of the high temporal resolution of our measurements and to gain more nuanced insights into the learning experience, we investigate whether the relationship between attention and knowledge is particularly pronounced during moments when relevant information is presented in the video. Additionally, we aim to explore the interaction effect time and post-knowledge have on GCM.

## Results

**Fig. 1:**
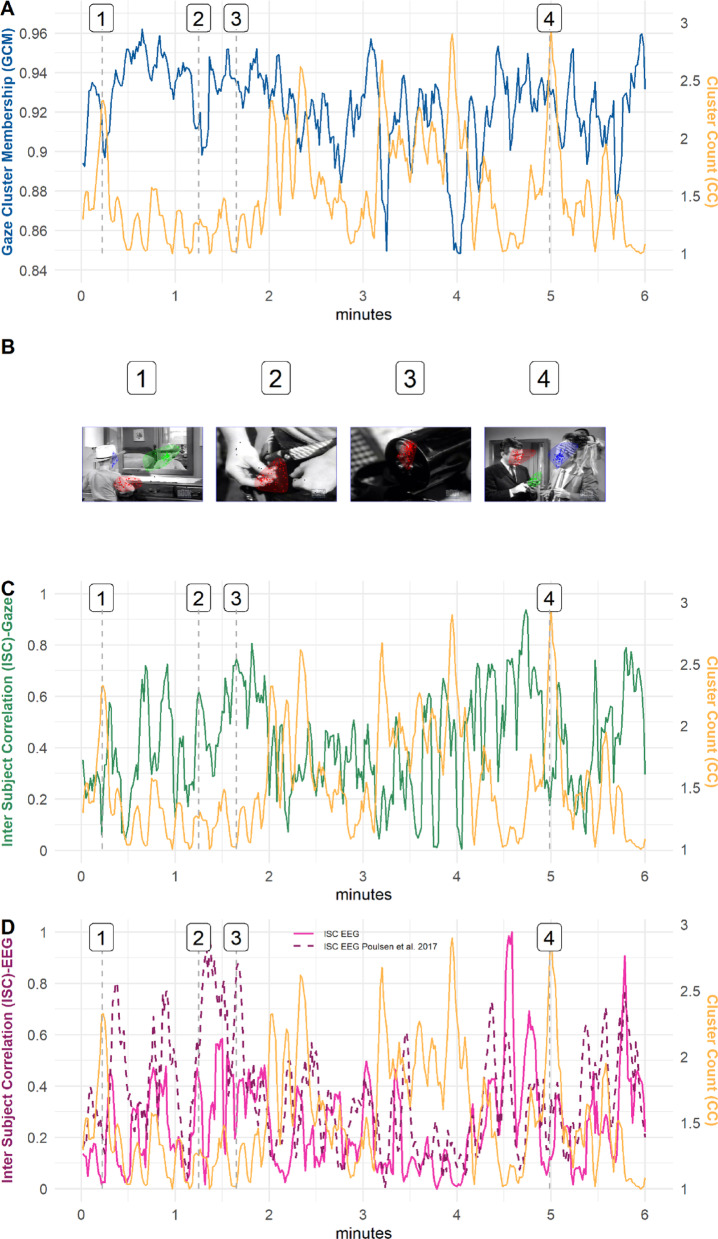
Comparison of the time course for cluster count (CC), gaze cluster membership (GCM) and inter-subject correlations (ISC_Gaze_, ISC_EEG_, ISC_Poulsen et al. 2017_) for the video clip “Bang! You’re Dead”. *Note*. GCM and cluster count were smoothed using a 5 s moving window with a 1 s interval. (**A**) Cluster Count and GCM over the time course of the video clip “Bang! You’re Dead”. (**B**) Example frames from the video clip “Bang! You’re Dead”. (1) high cluster count, low GCM and low ISC values; (2) low cluster count, low GCM and high ISC values; (3) low cluster count, high GCM and high ISC values; (4) high cluster count, high GCM and low ISC values. (**C**) Cluster Count and ISC_Gaze_ over the time course of the video clip “Bang! You’re Dead”. (**C**) Cluster Count and ISC_EEG_ (pink) and ISC_EEG (Poulsen et al. 2017)_ (magenta, dotted line) over the time course of the video clip “Bang! You’re Dead”.

**Fig. 2:**
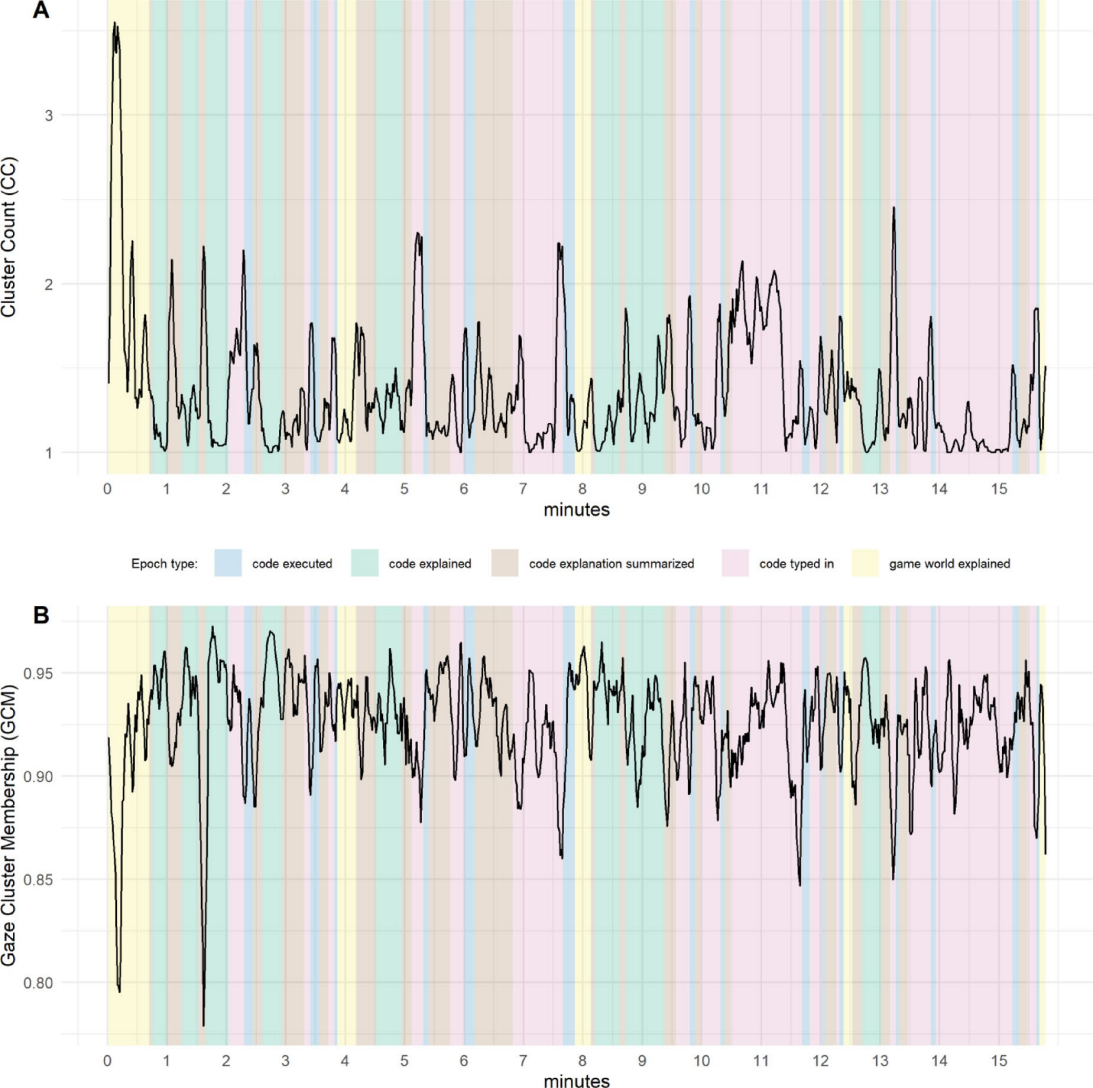
Comparison of the time course for cluster count (CC) and gaze cluster membership (GCM) with periods of interest (POI) for the instructional video “Programming in Minecraft”. *Note*. GCM and cluster count were smoothed using a five seconds moving window with a one second interval. Cluster count (**A**) and GCM (**B**) over the time course of the video “Programming in Minecraft” with periods of interest in the background.

For the 8630 video frames (360 s; fps = 23.98) for the video clip “Bang! You’re Dead” a total of 13,189 clusters were detected, with one to six clusters per video frame. Participants’ gaze was on average 94% (*SD* = 0.10) of the time within a cluster (i.e., belonging to one of the potentially several clusters and classified as attentive viewing; see Fig. 1A). For the 23,664 video frames (946.56 s; fps = 25) in the instructional video “Programming in Minecraft”, a total of 31,366 clusters were detected, with zero to seven clusters per video frame (see Fig. 2A). Participants were on average 92% (*SD* = 0.13) of the time in a cluster (see Fig. 2B). Tables 1 and 2 in the Appendix give an overview of the correlations among all variables for both videos. All models described below can be found in Tables 3 and 4 in the Appendix.

### Ratings for meaningfulness of clusters

To demonstrate that the automatic cluster detection with DBSCAN reliably detects gaze clusters that provide relevant information (i.e., for learning or for following the story), the meaningfulness of the content shown in these clusters was manually verified by human raters. For the video clip “Bang! You’re Dead”, only 317 out of the 13,189 clusters (2.40%) were rated as not meaningful. For the instructional video “Programming in Minecraft”, only 970 out of 31,366 clusters (3.09%) were found in regions that did not convey meaningful information, indicating that for both videos the vast majority of automatically identified clusters conveyed meaningful information.

### Comparing GCM and ISC in (multi-foci) scenes

To illustrate the problem of single-focus measurements in multi-foci scenes and the advantages that GCM has over single-focus approaches, we contrasted GCM and ISC (assessed using both neuronal and eye tracking data) in single and multi-foci scenes from a video clip of “Bang! You’re Dead”. The measure cluster count (CC) reflects the number of foci present in each scene. The scenes in the video have been previously rated for their levels of suspense in other studies^38,39^. We used two scenes of high suspense (single focus scene of a close-up when the boy loads the gun with a real bullet (3); multi-foci scene when adults find out the boy has a real gun (4)) and two scenes of low suspense (single focus scene when the boy puts bullets in his pocket (3); multi-foci scene of the boy putting things away in the cupboard (1); see Fig. 1B). Figure 1A shows that for GCM in scenes with multiple foci as in frame (1) and (4), it is possible that GCM is rater low (1), indicating inattention as it is a scene of low suspense, or rather high (4), indicating attention as it is a scene with high suspense. This shows that even in multi-foci scenes it is possible for GCM to differentiate between attention and inattention. This is however not the case for the ISCs (ISC_Gaze_, ISC_EEG_, ISC_Poulsen et al. 2017_); in both frames (1) and (4) the ISCs values are rather low, suggesting inattention and thus demonstrating the inability to differentiate between attention and inattention in multi-foci scenes. The ISCs values are rather high in scenes with only one focus, as in frames (2) and (3), indicating attentive participants, but frame (2) is a scene with low suspense (see Fig. 1C,D).

### GCM and mental effort

GCM as an indicator of attention over the entire video should be associated with the attention related construct mental effort. Therefore, after watching the “Programming in Minecraft” video, participants rated their mental effort while viewing it. A linear regression model (including covariates; see Table 3 in the Appendix for more information) revealed that GCM significantly predicted mental effort, *t*(102) = 4.02, *p* < 0.01, β = 0.38.

### GCM and knowledge

To explore whether GCM predicted learning, participants’ knowledge regarding the instructional video “Programming in Minecraft” was assessed both before and after viewing the video. In a first step, we ran a linear regression model (including covariates, see Table 3 in the Appendix) that revealed that GCM significantly predicted participants post-knowledge over the entire video, *t*(101) = 2.56, *p* = 0.01, β = 0.23.

**Fig. 3:**
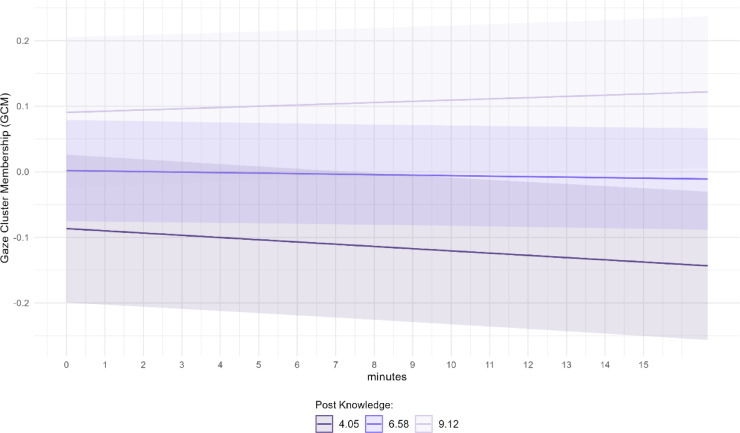
Predicted values of gaze cluster membership (GCM) over the time course of the video “Programming in Minecraft” . *Note.* GCM was smoothed using a five seconds moving window with a one second interval and z-standardized. GCM over the time course of the video “Programming in Minecraft” for three groups (high, medium, low post-knowledge), including the groups confidence intervals.

In order to take advantage of the high temporal resolution of our measurement and to gain more nuanced insights into the instructional video and GCM, we transcribed and categorized the video content into five distinct periods of interest (POI). These included one period that coded times when non-programming content (i.e., game world explained) was taught and was therefore irrelevant to knowledge acquisition, and four periods that represented times when programming-related content was taught (i.e., code explained, code explanation summarized, code typed in, code executed; see Fig. 2A) and was therefore relevant to knowledge acquisition. We estimated a linear mixed effects model and used GCM as the dependent variable. The model incorporated fixed effects at Level 2, which included the participant-level variables prior-knowledge and post-knowledge. At Level 1, we included POI as a predictor, with the irrelevant period as the reference category, along with time as another predictor. We examined interaction effects between post-knowledge and POI, as we expected that post-knowledge should only be related to GCM in POI of programming-related content. Additionally, we explored the interaction effect of post-knowledge and time. To account for individual differences among participants, we included a random intercept for each participant in the model. The results revealed significant interaction effects for post-knowledge with POI and time. Specifically, GCM was predicted by the interaction of post-knowledge with the following periods of interest: code explained, *t*(92) = 6.56, *p* < 0.001, β = 0.08, code typed in, *t*(92) = 10.17, *p* < 0.001, β = 0.12, and code executed, *t*(92) = 3.61, *p* < 0.001, β = 0.05. A trend that reached no significance was found for the category code explanation summarized, *t*(92) = 1.92, *p* = 0.06, β = 0.02. These results show that the relationship between attention and learning is especially pronounced at moments when relevant knowledge is presented in the video. There is also a significant interaction effect for post-knowledge and time, *t*(92) = − 3.47, *p* < 0.001, β = 0.01, showing that the gaze of participants with the lowest post-knowledge drop out of clusters more over time than the gaze of participants with higher post-knowledge (see Fig. 3).

## Discussion

The current study introduced a new eye tracking measure labeled gaze cluster membership (GCM) as an indicator for attention while watching videos and demonstrates its applicability for an instructional video. As attention plays a crucial role in the learning process^5^, an obvious implication for the effective design of such videos is to include elements that promote prolonged attention. The underlying assumption of GCM is that not necessarily one single but rather multiple meaningful focal points (i.e., different viewers focus their attention on different areas of a scene) can reflect attentive viewing. Consequently, we used a cluster detection algorithm (i.e., DBSCAN) that grouped gaze position data into clusters at a single video frame level. Gaze positions of an individual belonging to one of the potentially several clusters were classified as attentive viewing. By contrast, inattentive viewing was detected when the gaze position of the individual could not be assigned to a cluster.

The application of an unsupervised machine learning approach for gaze cluster detection offers several advantages. GCM eliminates the need to pre-define AOIs as regions that convey meaningful content^13,14^. Not only is the creation of AOIs time consuming, but it also restricts the gaze measure of attention to a selected set of options, which can introduce researcher- or theory-driven biases. The negligible number of clusters that were rated as not conveying meaningful content (< 4%) by rater(s) in two very different videos (a short film and an instructional video) indicates a high level of detection validity and the applicability of the DBSCAN settings to create clusters that convey meaningful content.

The development of GCM was driven by our skepticism towards approaches such as ISC that assume single meaningful focal points. And indeed, a direct comparison of ISC with GCM and its accompanying measure cluster count show that ISC provides an incomplete picture of attention in high suspense scenes where more than one region contains meaningful information. Namely, low ISC values (i.e., greater dissimilarity of neural signals or gaze patterns) typically interpreted as inattentive viewing are associated with higher levels of cluster count. Higher cluster count values, however, do not necessarily indicate inattentiveness for as long as the viewers’ gaze position belongs to one of the several clusters (i.e., high GCM values). Therefore, we would argue that GCM allows a more appropriate distinction between attentive and inattentive viewing. Our results do not contradict findings that ISC can predict knowledge^18,33^ we just demonstrate the limitations of ISC’s interpretability concerning attention in scenes where more than one region contains meaningful information. This limitation of the ISC’s interpretability concerning attention is not only for the ISC of gaze data, but also for the neuronal data (i.e., ISC_EEG,_ ISC_Poulsen et al. 2017_) and is consistent with existing research showing that different ISC measures are correlated with each other^32^. An innovative aspect of the current study was the use of cEEGrids instead of EEG caps to capture the neuronal signals, hence we also compare a digitized time course plot (ISC_Poulsen et al. 2017_) alongside with our ISC_EEG._ The limitation of the ISC’s interpretability holds true for both neuronal ISCs, demonstrating that the results are not dependent on the method of data acquisition (EEG caps vs. cEEGrids).

Additional evidence for the validity of GCM as an indicator for attention is provided by the result that GCM predicted self-reported ratings of mental effort (i.e., how hard a person tries to actively process presented information^37^). The initial motivation for GCM’s development was the need for a valid measure of attention, that can predict learning progress. As attention is crucial in the process of knowledge acquisition^5,6^ GCM could be used to improve the quality and efficiency of instructional videos. The result of the linear regression model, that higher GCM—controlled for prior-knowledge—predicts posttest knowledge illustrates the diagnostic relevance of GCM in educational contexts. In addition, GCM’s high temporal resolution provides valuable insights into attention effects related to video content in specific time windows, as well as the effect of time on attention. The linear mixed effect model with the predictors POI and time and their interaction with posttest knowledge demonstrated that GCM was indicative in periods provided crucial information for knowledge tests in contrast to a period with content irrelevant for posttests.

Furthermore, the interaction of time and posttest knowledge indicates a general decrease of GMC over time. The decline in attention over time during lectures is in line with other studies on attention that have used self-report measures ^e.g.40^. The decrease is especially pronounced for the gaze of participants with lower posttest knowledge. This highlights the critical importance of re-engaging viewers, because as time progresses, the situation worsens for these learners. To address this, incorporating strategies to re-engage learners (e.g., interactive quizzes or polls) or providing them with the option to revisit previous sections of the video, could be beneficial.

### Limitations and further research

Although the current study tested GCM on two very different types of videos (a short film and an instructional video), this newly introduced measure requires replication in general, in other areas of education, and with longer video material. The latter is especially relevant to test GCM’s applicability in real-world settings. An important limitation of the current study in this context is that data were collected in a highly controlled laboratory setting. The use of a chinrest—to minimize head movements—affected participants’ comfort and created a rather artificial situation, which might have affected participants’ attention and general compliance. A more realistic setting might be achieved through webcam based eye-tracking^18^ at participants’ homes. A direction for further investigations might be to let viewers pause and repeat parts of a video at their own pace, as this would correspond to typical behavior that also takes the advantages of online videos into account^10^.

Additional research is necessary to further differentiate between effortful versus effortless attention control, as well as to identify inattentive viewers who may not perceive the video content as challenging, or the information presented as not novel enough. While we use the term attention for GCM, it could be any internal state of an individual that might influence knowledge and eye movements, including alertness, interest, motivation, engagement, and fatigue.

Like other measures, GCM could be manipulated by individuals. If enough participants simultaneously focus on an irrelevant point, they create a cluster that inaccurately labels them as attentive, even though they are not truly engaged. Furthermore, GCM encounters a similar flaw as AOIs regarding the percentage of screen area covered by clusters. When clusters occupy a larger portion of the screen, the statistical likelihood of being in a cluster increases. Additionally, if there are captivating but distracting elements in the instructional video that draw participants’ attention GCM may not be predictive for knowledge. When attention is diverted towards these seductive details, essential information needed for learning may not be adequately processed, as these distractions could overload working memory and lead to attention diversion^41,42^.

It is difficult to give clear recommendations for the minimum number of participants required, as the characteristics of a video and individual differences in viewers will affect the reliability of cluster detection. Based on preliminary testing, we recommend at least 50 participants to ensure reliable clusters and GCM values. Future studies might also combine GCM and AOIs by generating AOIs based on the clusters detected for a sufficient number of participants. This approach could involve applying these AOIs to a different dataset that uses the same video, or alternatively if the dataset is large enough, splitting the data into two subsets—that is, creating clusters from one subset and then applying the AOIs to investigate attention during video viewing for the newly added individuals of the other subset.

## Conclusion

In conclusion, we have developed a new gaze-based high temporal measure of attention, GCM, with the theoretical underlying assumption that a scene can have more than one meaningful focal point. We found that GCM was predictive of participants’ mental effort and knowledge. This innovative approach for the estimation of attention at a high temporal resolution holds great promise for future research and various applications. It allows for a deeper analysis of attention while watching video content and can enhance instructional design as instructors can use this information to identify challenging sections of videos and pinpoint moments when learners’ attention wanes.

## Method

### Sample

A convenience sample of 121 adults (age *M* = 25.76 years, range 18–55, *SD* = 7.41, 67% female) participated in the study and gave written informed consent prior to their participation. Ethical approval for this study was obtained from the Department 05 Ethics Committee at Goethe University Frankfurt and performed in accordance with the Declaration of Helsinki. Sample sizes differed for the two videos (i.e., “Bang! You’re Dead” and “Programming in Minecraft”) and the two measurement types (i.e., eye tracking and EEG) due to varying reasons, which are listed below.

In the video condition “Bang! You’re Dead”, the eye tracking data of one participant were rejected due to an incomplete recording, and the data of eight participants were excluded because of poor data quality (for details on poor data quality criteria, see the sections on data acquisition and preprocessing). Therefore, the final sample comprised 112 participants with eye tracking data. EEG data of four participants were rejected due to an incomplete recording and data of one participant was excluded because of poor data quality. The final EEG sample comprised 116 participants.

In the video condition “Programming in Minecraft”, eye tracking data for four participants were unavailable due to technical issues, data of one participant was rejected due to an incomplete recording and eleven participants were excluded from further analysis due to low data quality. This resulted in an eye tracking sample of 105 participants.

### Video stimuli

Two videos were used in this study: one is a snippet of 6:00 min of the short film “Bang! You’re Dead” directed by Alfred Hitchcock (1961), covering a section from 06:28 to 12:28 in the full movie. We chose this video, because of its high levels of suspense and tension^38,39,43^ and its usage in previous ISC studies^44,45^. The narrative centres around a young boy who unwittingly holds a genuine revolver loaded with a single bullet, which is triggered at various points throughout the video, creating an atmosphere of intense anticipation for the audience. The second video is a self-produced instructional video, which provides an introduction to the programming language Python and was set up in a Minecraft Education environment. This video explores the fundamentals of writing code and describes how the syntax is structured in Python. The “Programming in Minecraft” video was 15:46 min long.

### Instruments

#### Mental effort

Mental effort was rated by participants after watching the “Programming in Minecraft” video with seven adapted items^46^. The items were rated on a 6-point Likert scale ranging from 1 (strongly disagree) to 6 (strongly agree). A sample item is “I have put little effort into watching the video”. The mean rating was calculated for each participant, and the scale’s internal consistency was good: Ω = 0.89^47^.

#### Knowledge test

Participants’ knowledge of basic programming in Python was assessed using a 13-item knowledge test. The test included seven multiple choice items, one drag-and-drop item, and five items with a free-response format. Responses for the latter were coded by two trained raters in a consensus rating. Seven of the 13 items were used in the pre-test to control for participants’ prior knowledge. All items were used in the post-test. Participants could receive up to one point per item and a sum score was calculated across all items in the pre- and post-test. A sample item is “What is the purpose of a loop in Python?” For the pre-test, internal consistency was rather low: Ω_post_ = 0.60^47^. Rather low internal consistencies are not unusual in performance assessments, especially if participants do not have much or diverse knowledge about a topic^48^. For the full post-test version, internal consistency was satisfactory: Ω_post_ = 0.78^47^.

### Procedure

Prior to the study, the skin around participants’ ears was de-greased using a cotton pad and an alcohol solution. Then, one drop of electrolyte gel (Abralyt, Easycap GmbH, Germany) was applied to each electrode of the cEEGrids (TMSi, Netherlands). CEEGrids are a C-shaped, unobtrusive electrode array with 20 printed electrodes (ten on each side) that are integrated into adhesive patches and placed around the participant’s ears^49^. Two cEEGrids were then affixed around each ear and connected to a mobile EEG device (SMARTING; mBrainTrain, Belgrade, Serbia) in the back of a headband worn by participants. To ensure good data quality, impedance was measured prior to recording using Smarting Streamer (version 3.4.3, mBrainTrain, Belgrade Serbia), balancing good data quality (i.e., below 20 kOhm) with a reasonable time limit that took participant comfort into account.

The study was conducted in a darkened room with a small light source to maintain consistent lighting conditions. The two videos were presented in full-screen mode on a 21-inch monitor (1680 × 1050). For both videos, participants’ eye movements were recorded from their right eye at a sampling rate of 500 Hz, using an EyeLink 1000 (SR Research Ltd. Ottawa, Canada). Participants’ heads were stabilized with a chin and forehead rest ensuring a 45 cm distance between eye and presentation monitor. Prior to watching the videos, participants were instructed to sit still. The eye-tracking system was calibrated before each video presentation using a standard five-point calibration.

In order to synchronize stimulus timing across participants, videos were presented using the open source software PsychoPy^50^, which sent event markers three seconds before video onset and three seconds after video offset. Participants saw a blank grey screen during this time. Event markers were sent via LabStreamingLayer (LSL)^51^ into the EEG data stream and simultaneously as messages into the eye tracking recordings.

Participants first watched the video “Bang! You’re Dead”. Before watching the instructional video “Programming in Minecraft”, participants filled out a short (approximately 10 min to complete) pre-test questionnaire on a tablet computer, assessing their prior knowledge about the programming language Python. After watching the instructional video, a post-test questionnaire (approximately 20 min to complete) assessed participants’ post knowledge and mental effort. The study was part of a larger experiment with two instructional conditions. We checked whether the results were related to the conditions, and as this was not the case, the conditions were not further included in the analyses. Participants received either financial compensation or university credit points.

### Data acquisition and preprocessing

#### EEG data acquisition and preprocessing

Raw EEG data were sampled at 250 Hz and streamed via Bluetooth to a presentation computer. Using LSL, data were streamed via a local area network connection to and stored on a separate computer in LSL’s xdf-format^51^. In a first step, EEG data and their associated event marker streams were extracted from xdf files and saved in MNE Python’s^52^ fif format. Using MNE Python^52^, raw EEG data were band-pass filtered between 0.5 and 45 Hz and re-referenced to the arithmetic mean of the original recording reference located behind the right ear and its counterpart on the left.

Starting at 2.5 s prior to video onset data were segmented into consecutive epochs of one second length. On a single electrode level, epochs were defined as bad if peak to peak changes in activity exceeded 100 μV. The complete epoch was marked as bad, if more than three channels were affected by this noise. Participants with more than 30% bad epochs were excluded from further analysis.

#### Eye tracking pre-processing

Raw eye tracking data were recorded in SR-Research’s binary edf-format and converted into an ascii-format. Based on an algorithm that detects abrupt changes in pupil size, blink on- and offsets were marked within gaze data. In addition, a 60 ms time window before and after each blink was also marked, which addressed the problem that blinks are—due to the closing and opening eyelid—preceded by a sudden downwards shift and followed by a sudden upwards shift of gaze positions. Gaze position data for the entire time window (60 ms before, the blink itself, and 60 ms after the blink) were re-coded as missing data. Also, gaze positions detected outside of the monitor’s dimensions were first marked as such, and in a next step re-coded as missing data.

The two criteria, “blink count” and “percentage of missing data”, normalized across participants, were used for outlier detection. That is, data from participants who scored higher than two standard deviations on at least one of the criteria were excluded from further analysis. As the correlated component analysis for the ISC gaze calculation cannot handle missing data, blinks were filled with linearly interpolated values.

### Data analysis

#### ISC of EEG and gaze

The ISC of the EEG data for “Bang! You’re Dead” was calculated using correlated component analysis, using the first component with the highest correlation between subjects. To compute the ISC for each component, correlation coefficients of the EEG are calculated between each participant and all other participants^53,54^. The same procedure was applied for the gaze data.

A novel aspect of the current study is the use of cEEGrids instead of traditional EEG caps to capture the neuronal signals. Therefore, in addition to our ISC_EEG_, we use ISC_Poulsen et al.2017_, a digitized time course plot of Figure 4^44^, using the web service Web Plot Digitizer^55^ to ensure, that the results are not compromised by our method of data acquisition.

#### Gaze cluster membership

Cluster detection using the DBSCAN algorithm^36^ was performed on a video frame time scale, that is, the video clip “Bang! You’re Dead” consisted of 8630 frames (23.98 fps × 360 s) and the “Programming in Minecraft” video of 23,664 frames (25 fps × 947 s). For each time window corresponding to one video frame (“Bang! You’re Dead” 41.7 ms; “Programming in Minecraft” 4.0 ms) gaze positions were averaged for each participant. The DBSCAN algorithm then grouped data into clusters of neighboring gaze positions and categorized each participant’s gaze as being a member of one of potentially several clusters or as an outlier (i.e., not belonging to any cluster)^35,36^. Two key parameters must be specified for the DBSCAN algorithm, that is, the minimum number of points needed to form a cluster and the maximum distance (ε) between two points to be considered as neighboring. The minimum cluster size was set to either 5% of the available data points (i.e., participants with at least on valid gaze position per frame) or at least 4 data points. Based on the k-nearest neighbor distances a suitable value for ε was select as the first value that exceeded one standard deviation. Since the resulting time series contained a lot of high frequent fluctuations, the data were smoothed by applying a five second moving window average for one second intervals.

#### Rater agreement for meaningfulness of clusters

To demonstrate that the automatic cluster detection with DBSCAN reliably detects gaze clusters that provide meaningful information, all detected clusters were rated as either meaningful (i.e., clusters of gaze positions found in regions that convey meaningful content for learning or for following the story) or not meaningful (i.e., clusters of gaze positions found in regions that do not convey meaningful information).

A latency of up to 250 ms for gaze shifts to new positions was taken into account^56^. In instances where the raters disagreed, a consensus decision was reached. The agreement between the two raters for the video clip “Bang! You’re Dead” was very high; in 8346 out of 8630 video frames (96.71%), both raters agreed for all clusters shown in the frame. Given the high rater agreement for the video clip “Bang! You’re Dead”, ratings were only obtained from one rater for the video “Programming in Minecraft”.

#### Video transcription “programming in minecraft”

The video was transcribed and divided into 72 segments of content units by one human rater. The segments were rated by two raters who examined each segment to determine its content category based on a manual that provided definitions and anchor examples. The periods of interest (POI) included one irrelevant to knowledge, labeled as “game world explained” (4 segments, 1:32 min) alongside four periods relevant to knowledge acquisition: “code explained” (9 segments, 3:04 min), “code explanation summarized” (23 segments, 3:58 min), “code typed in” (21 segments, 5:43 min), and “code executed” (15 segments, 1:30 min). In cases of disagreement between the raters, a consensus rating was used to reach a decision.

### Statistical analyses

Analyses were performed in R (version 2023.12.1, R Core Team, 2023). Significance tests were performed at the 0.05 level. Continuous variables were transformed into z-scores for standardization.

To ensure the comparability of GCM, ISC_EEG_, and ISC_Gaze_, only those participants for whom both data sets were available are included (N=110). To investigate whether GCM predicts mental effort, a linear regression model was conducted with mental effort as the dependent variable while controlling for missing gaze data. To investigate whether GCM predicts knowledge, we first performed a linear regression model using post knowledge as the dependent variable while controlling for missing gaze data and prior knowledge. Finally, a linear mixed effects model was employed with GCM as the dependent variable, including POI (irrelevant POI as reference group), time, prior-knowledge and post-knowledge as fixed effects. Additionally, interaction effects between post-knowledge and POI, as well as between post-knowledge and time, were included. Variability among participants was accounted for by including a random intercept for each participant.

## Data Availability

Data and scripts supporting this study are openly available in the Dataverse repository, https://osf.io/4rn5q.

## Appendix

See Tables 1, 2, 3 and 4.

**Table 1:** Zero-order correlations among all variables for the video clip “Bang! You’re Dead”.

	*M*	*SD*	01
01 Gaze cluster membership (GCM)	0.92	0.06	
02 Gaze missing	0.06	0.05	− 0.39*

**p* < 0.05.

**Table 2:** Zero-order correlations among all variables for the video “Programming in Minecraft”.

	*M*	*SD*	01	02	03	04
01 gaze cluster membership (GCM)	0.92	0.05				
02 Gaze missing	0.08	0.05	− 0.18			
03 Mental effort	4.14	0.86	0.38*	− 0.07		
04 Knowledge about programming (post)	6.56	2.57	0.20*	− 0.04	0.28*	
05 Knowledge about programming (pre)	1.87	1.21	− 0.07	− 0.03	0.14	0.39*

**p* < 0.05.

**Table 3:** Regression model results for the outcomes for the video “Programming in Minecraft”.

	*b*	*SE*	*t*	*p*	*R* ^*2*^
Mental effort					0.12
(Intercept)	4.14	0.08	53.02	< 0.001	
Gaze cluster membership (GCM)	0.32	0.08	4.02	< 0.001	
Gaze missing	0.00	0.08	− 0.05	0.96	
Knowledge about programming (post)					0.19
(Intercept)	6.54	0.23	29.03	< 0.001	
Gaze cluster membership (GCM)	0.59	0.23	2.56	0.01	
Gaze missing	0.04	0.23	0.18	0.86	
Knowledge about programming (pre)	1.07	0.23	4.70	< 0.001	

*Note*. The predictor variables in the linear regression models were z-standardized.

**Table 4:** Linear mixed-effects model for GCM for the video “Programming in Minecraft”.

Fixed effects	*b*	*SE*	*t*	*p*
(Intercept)	− 0.04	0.04	− 1.09	0.28
Label code explained	0.14	0.01	12.30	< 0.001
Label code executed	0.01	0.01	0.64	0.52
Label code typed in	− 0.01	0.01	− 1.22	0.22
Label code explanation summarized	0.06	0.01	5.15	< 0.001
Knowledge about programming (post)	0.04	0.04	1.00	0.32
Time (sec)	− 0.00	0.00	− 1.11	0.27
Knowledge about programming (pre)	− 0.07	0.04	− 1.67	0.10
Label code explained × Knowledge about programming (post)	0.08	0.01	6.56	< 0.001
Label code executed × Knowledge about programming (post)	0.05	0.01	3.61	< 0.001
Label code typed in × Knowledge about programming (post)	0.12	0.01	10.17	< 0.001
Label code explanation summarized × Knowledge about programming (post)	0.02	0.01	1.92	0.06
Time (sec) × Knowledge about programming (post)	0.01	0.00	3.64	< 0.001

Random effects		Variance	Std.dev.	
Participant (Intercept)		0.16	0.40	
Residual		0.84	0.91	

*Note*. The predictor variables in the linear mixed effects model were z-standardized. The marginal R-squared (R^2^m) is 0.02, while the conditional R-squared (R^2^c) is 0.17.

*Std.dev.* standard deviation.
